# The Buffalo Obstetrical Society

**Published:** 1888-05

**Authors:** 


### *THE BUFFALO OBSTETRICAL SOCIETY.*

This society, organized only a few years ago, has accomplished excellent work, and the reports of its proceedings have been read with great interest. Upon the occasion of the meeting of distinguished obstetricians and gynecologists for the organization of a national association, the society demonstrated that, when "on hospitable thoughts intent," it could make a reputation on other than purely scientific lines. It having come to the knowledge of the members of the society that such a gathering of eminent men was about to convene in this city, it was determined to give them a fraternal greeting, and Prof. Thomas Lothrop was asked to express the welcome of the local society, which he did in the following words:

Gentlemen: On this auspicious occasion, which calls together from widely distant sections of our common country, representative men with a view to advance the interests of a great profession, I am bidden by my confreres of the Buffalo Obstetrical Society, and by members of the profession in our city, to tender you a cordial welcome.

It is a notable feature of our day that, in the various departments of human knowledge, associated effort in special lines of thought and study is adopted to assist the labors of scientific men. The organization of societies to promote the interests of other departments of medical science, by enlisting the energy and ability of men of like professional tastes and habits, have contributed in a great measure to the activity observed in professional circles on medical and surgical subjects. In the department of obstetrics and its allied science, gynecology, there is a wide field of labor, and of the most absorbing interest. If wisely directed, your efforts in organizing an association for the advancement of these special departments of medical science will bring forth results to bless humanity and the noble profession to which we belong. We rejoice at these evidences of interest and activity which have summoned you from your busy work, and for the good of the profession and humanity, to take sweet counsel together for the perfecting of your scientific labors.

Let not, however, your earnestness in organization, nor your zeal in this special department of medicine, hinder you from taking even a cursory view of our beautiful city, which, with its parks and boulevards, its educational and eleemosynary institutions, place it in a commanding position in the Empire State. While yet young in years, compared with the great centers of population of the East, Buffalo maintains a proud position by its increase in wealth and population, and in the advantages it offers for commerce and enterprise, inferior to none of her sister cities. The medical profession, for which I speak, has worthily maintained her reputation, and promoted, by honorable labor, her interests.

I beg to express the wish that your deliberations here will be fruitful of good in the special work which calls you from your distant homes; that your tarry in our city may be among the pleasant incidents in your busy and useful lives; that your stay may be prolonged to enjoy the hospitality it is our privilege to tender you, and that we may improve the opportunity to become personally acquainted with our honored guests and also to be known better of them.

The limited time which the organizers of the Association had allowed themselves for a stay in Buffalo, made any formal gathering of the profession impossible, but it was determined that the society should at least meet their friends from abroad; consequently, during the midday intermission in their labors, a luncheon was spread at "The Niagara," and the members of the association and the Buffalo Obstetrical Society, together with a few invited guests from the city, sat down to a table which, with its decorations of flowers, fruit, the accompanying sweet music and the perfection of the menu offered, left nothing to be desired, at least in the estimation of the writer, who had the honor to be one of the invited guests. The absence of formality, necessary from the limited time at disposal, was rather a charm than otherwise, and the luncheon very happily ended with a short, off-hand speech from Prof. Thomas Opie, M. D., of Baltimore, in which he very humorously and eloquently returned the thanks of the association for the kind welcome and fraternal greeting received at the hands of the members of the Buffalo Obstetrical Society.

---

An extract from a lecture of Dr. William Murrell, of London, is printed in our advertising pages. He calls attention to the necessity of clearly indicating the variety of Pepsin when prescribing this important agent. Our own experience has led us to about the same conclusion as to the value of relative brands. *Fairchild's Pepsin* has never failed to give us satisfactory results. We have found it better than English, French, German or other pepsins. When a combination of the digestive agents is indicated, we use *Lactopeptin* with uniformly good effect.

